# Severe and Intractable Hypokalemia in a Patient With New-onset Type 1 Diabetes and COVID-19 Infection

**DOI:** 10.1210/jcemcr/luaf151

**Published:** 2025-07-11

**Authors:** Åke Sjöholm, Anna Bandert

**Affiliations:** Department of Internal Medicine, Division of Endocrinology and, Diabetology, Gävle Hospital, University of Gävle, Gävle SE-80324, Sweden; Department of Anesthesiology and Intensive Care, Gävle Hospital, Gävle SE-80324, Sweden

**Keywords:** type 1 diabetes, COVID-19, SARS-CoV2, hypokalemia, angiotensin-converting enzyme 2, aldosterone

## Abstract

A 29-year-old man was admitted to the hospital in a state of reduced consciousness with new-onset diabetes and positive for coronavirus disease 2019. He presented with severe ketoacidosis and profound hypokalemia. While his diabetic ketoacidosis was promptly corrected, it proved extremely difficult to maintain normokalemia. For a total of 49 hours, not less than 1127 mmol of i.v. Addex-potassium (potassium hydroxide and dipotassium phosphate trihydrate; ∼ 25 mmol/hour) in addition to 76 mmol/d of oral potassium was required, ie, tantamount to doses used to achieve cardioplegia. Severe, sustained, treatment-resistant and potentially lethal hypokalemia may thus occur in patients with severe acute respiratory syndrome coronavirus 2 (SARS-CoV-2) infection and should be actively monitored. Based upon these findings and converging evidence in the literature, we propose a model in which disruption of angiotensin-converting enzyme 2 by SARS-CoV-2 activates the angiotensin-II pathway, thereby enhancing aldosterone production. Excess aldosterone activates renal epithelial sodium channels, thus promoting massive loss of potassium through urinary excretion. This implies that severe hypokalemia by SARS-CoV-2 infection may be amenable to treatment with potassium-sparing drugs antagonizing the aldosterone receptor, such as spironolactone or eplerenone, whereas potassium supplementation even in very high doses may be futile.

## Introduction

The coronavirus disease 2019 (COVID-19) pandemic has profoundly and globally affected human health and disease panorama. While early therapeutic efforts focused on improving respiratory dysfunction, and rightfully so, it is now evident that infection with severe acute respiratory syndrome coronavirus 2 (SARS-CoV-2) also adversely influences metabolism and vascular function [1, 2].

This appears to be a bidirectional relationship: in the early phase of the COVID-19 pandemic, it became increasingly clear that patients with diabetes and/or obesity were found to be at increased risk to be infected by SARS-CoV-2 and had more complications and increased mortality compared to nonobese, nondiabetic individuals [1-3].

Conversely, early in the COVID-19 pandemic, case studies reported new-onset diabetes, both type 1- and type 2-like, in close connection with the SARS-CoV-2 infection [4-6]. However, it cannot be excluded that these persons would have developed diabetes anyway and that it just happened to coincide with the SARS-CoV-2 infection. Systemic reviews have shown conflicting results as to whether there is any increased risk of new-onset diabetes after SARS-CoV-2 infection [1-3], and this remains an open question subject to much debate.

In the quest to understand the precise mechanisms by which SARS-CoV-2 enters and invades cells, it was discovered that 1 such protein is angiotensin-converting enzyme 2 (ACE-2), which is disrupted by the virus [7]. ACE-2 is an integral part of the renin-angiotensin-aldosterone system (RAAS) that not only controls electrolyte balance, vascular tone, and blood pressure but also is highly expressed in the kidney, particularly in the proximal tubules. ACE-2 is also abundantly expressed in tissues relevant to diabetes, eg, pancreatic islets [8-10], and regulates islet blood flow [11].

## Case Presentation

**Figure 1.**
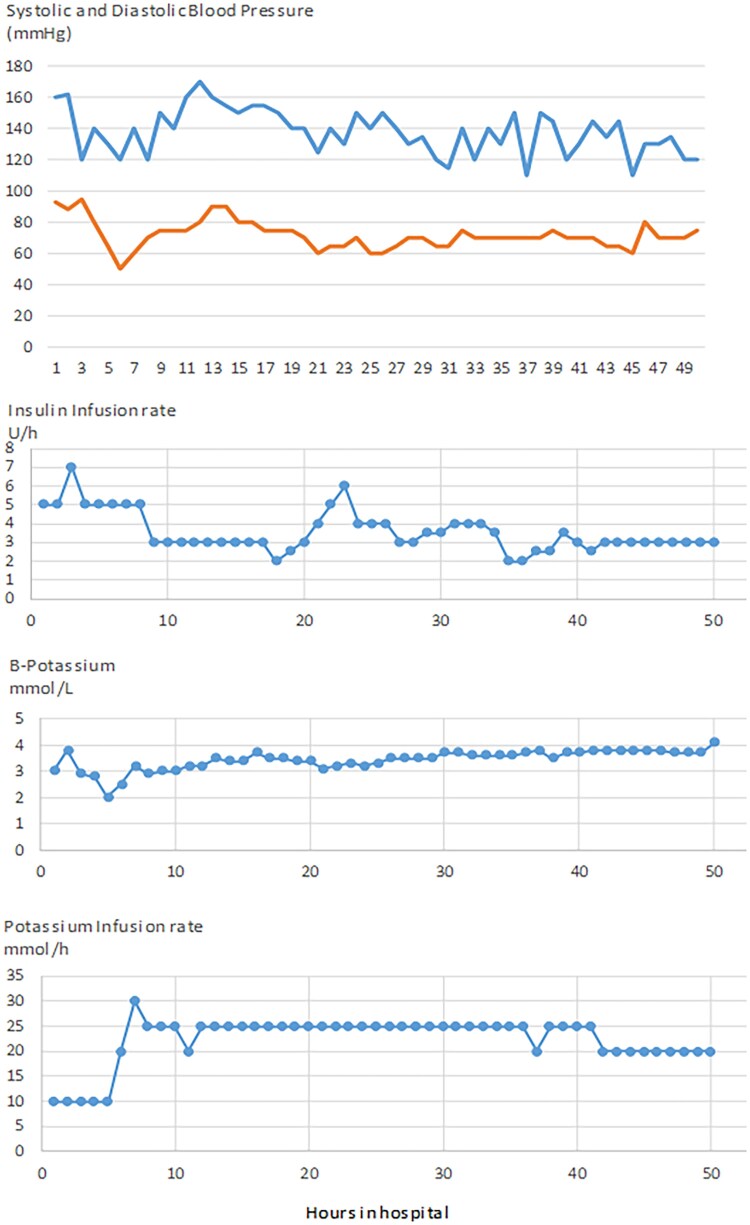
Time course showing blood pressure (blue, systolic; red, diastolic), insulin infusion rates, blood potassium levels, and the amount of IV potassium supplementation given during the patient's stay in the hospital.

A 29-year-old male without any significant previous medical history and no current medications was admitted to the emergency department due to impaired consciousness. He had complained of abdominal pain, nausea, polydipsia, and polyuria for a few days, and on the day of admittance his mother found the patient unconscious and unresponsive to pain stimulation. In the emergency department, the patient presented with impaired consciousness (reaction level scale ∼ 3), tachypnea (36 breaths/minute), tachycardia (100 beats per minute), and hypothermia (34.6 °C [94.3 °F]) but not hypotension (see Fig. 1). Upon admission, the patient was obtunded and drowsy but responded to light verbal stimuli. There were no focal neurological deficits, and no cranial computed tomography scan was performed.

## Diagnostic Assessment

An arterial blood gas analysis showed pH 6.79 (reference range 7.35-7.45), pO_2_ 23.8 kPa (reference range 10-11 kPa), pCO_2_ 1.8 kPa (reference range 4.8-5.8 kPa), BE −32 mmol/L (reference range −3-3 mmol/L), bicarbonate 12.2 mg/dL (SI: 2 mmol/L [reference range 134-159 mg/dL; SI: 22-26 mmol/L]), potassium 2.0 mmol/L (reference range 3.5-5.5 mmol/L), glucose 558 mg/dL (SI: 31 mmol/L [reference range 75-113 mg/dL; SI: 4.2-6.3 mmol/L]), lactate 13.5 mg/dL (SI: 1.5 mmol/L [reference range 6.3-22 mg/dL; SI: 0.7-2.5 mmol/L]), and ketones 40.6 mg/dL (SI: 3.9 mmol/L [reference range <15.6 mg/dL; SI:<1.5 mmol/L]), thus a severe diabetic ketoacidosis. The patient was also positive for SARS-CoV2 by PCR and antigen analyses. Routine blood work ups were essentially unremarkable. The level of glycated hemoglobin (HbA_1c_) was 15% (SI: 140 mmol/mol [reference range 2.9-4.5%; SI: 27-42 mmol/mol]), indicating a pronounced hyperglycemia.

The patient was assessed as hypovolemic at the time of admission, attributed to osmotic diuresis secondary to hyperglycemia. Serum osmolality on arrival was 315 mOsm/kg (reference range: 280-300 mOsm/kg), 311 mOsm/kg on the following day, and 316 mOsm/kg on the third day.

Regrettably, serum aldosterone levels or plasma renin activity were not measured. This patient was hospitalized in early January 2021, when the COVID-19 pandemic was relatively new and the disruptive effects of SARS-CoV2 on ACE-2 were not widely known at the time. Since then, mechanistic studies have compellingly shown such a link [9, 12, 13]. Had this patient presented today, we definitively would have measured serum aldosterone levels and plasma renin activity and attempted to correct his hypokalemia with potassium-sparing drugs.

## Treatment

In the emergency department, the patient was infused with 2 liters of Ringer Acetate to which 40 mmoles of Addex-potassium (potassium hydroxide and dipotassium phosphate trihydrate) were added. He also received 14 units of insulin aspart subcutaneously before immediate transferal to the intensive care unit (ICU).

In the ICU the patient was put on IV insulin infusion (initially 5 U/hour; see details over time in Fig. 1) and thiamine 100 mg once daily and received a total of 11 liters of crystalloids over 2 days. He did not require any inotropic support and swiftly normalized both glycemia and ketonemia. However, his hypokalemia was pronounced and persisted despite very high doses of IV potassium supplementation in addition to 76 mmol/day of oral potassium citrate. The need for extra potassium was so great that it necessitated insertion of a central venous catheter as the route of potassium treatment, but despite IV infusion of ∼ 25 mmol/hour (ie, ∼ 600 mmol/day) of Addex-potassium over 2 days in the ICU, it was very difficult to achieve normokalemia (plasma potassium > 3.5 mmol/L). For a total of the 49 hours in the ICU, not less than 1127 mmol of IV Addex-potassium was required. The time course of plasma potassium levels and the amount of IV potassium supplementation during the patient's stay in the ICU is shown in Fig. 1.

Admittedly, the patient's hypokalemia did improve after some time; however, this required exceedingly high (cardioplegic) doses of IV potassium supplementation. We interpret this delayed improvement as an effect of the RAAS recovering during the natural course of the SARS-CoV-2 infection. Since people infected with SARS-CoV-2 do not develop chronic hypokalemia of this magnitude, it is reasonable to view the RAAS disruption as a transient phenomenon.

A spot check of urine potassium showed a level of 45.4 mmol/L (reference < 20 mmol/L); unfortunately, no 24-hour collection was done. The patient did not receive any antiviral or dexamethasone therapy, as such treatment was not implemented in clinical practice at the time. He also did not require supplemental oxygen therapy at any time.

Plasma creatinine levels were mildly elevated at 1.53 mg/dL (SI: 135 µmol/L [reference range: 0.68-1.19 mg/dL; SI: 60-105 µmol/L]) upon admission, peaking at 1.66 mg/dL (SI: 147 µmol/L) on day 3. Levels normalized 1 month after hospital discharge. The transient elevation was considered to be due to prerenal hypoperfusion. Renal ultrasound with Doppler imaging was performed on day 6 and revealed no pathological findings.

After 2 days in the ICU, the patient was transferred to a regular medical ward and the IV insulin infusion was discontinued and replaced by a basal-bolus subcutaneous. insulin regimen (insulin glargine 12 U once daily and mealtime insulin lispro [4-8 U 3 times per day]). At discharge from the hospital, potassium levels and blood pressure were normalized.

## Outcome and Follow-up

When revisiting the diabetes outpatient clinic 6 weeks later, the patient had a body mass index of 28.3 kg/m^2^ and an HbA_1c_ level of 10.9% (SI: 96 mmol/mol), and he was normokalemic and normotensive. His body mass index at presentation was 24.2 kg/m^2^. He was negative for autoantibodies against zinc transporter 8 and islet antigen 2 but positive for glutamic acid decarboxylase 65-kilodalton isoform (98 U/mL [reference < 5 U/mL]) and showed a weak insulin production with a fasting basal level of C-peptide of 0.88 ng/mL (SI: 0.29 nmol/L) that rose postprandially to 1.81 ng/mL (SI: 0.6 nmol/L [reference range 1.12-3.6 ng/dL; SI: 0.37-1.5 nmol/L]). His family history included several cases of type 1 diabetes on the maternal side and also in the patient's son. The patient was diagnosed with autoimmune type 1 diabetes.

The patient continued his multiple daily injection insulin therapy and revisited our outpatient diabetes clinic regularly. At his latest revisit in 2024, he had switched to continuous subcutaneous insulin infusion using an Omnipod 5 insulin pump connected to a Dexcom 6 continuous glucose monitor. His glycemic control at that time was excellent with an HbA_1c_ level of 4.9% (SI: 43 mmol/mol), time-in-range 75%, and mean P-glucose 122 mg/dL (SI: 6.8 mmol/L) with a SD of 50 mg/dL (SI: 2.8 mmol/L) over 14 days.

## Discussion

We report a case of new-onset type 1 diabetes with severe and treatment-resistant hypokalemia in a young patient with concomitant SARS-CoV-2 infection. One can only speculate as to whether there was any cause-and-effect relationship between the viral infection and the onset of diabetes and/or hypokalemia in this patient. Suffice to say, however, that there is a growing body of findings providing a theoretical framework with enticing links to such a possibility.

ACE-2 is abundantly expressed in renal proximal tubules controlling sodium and potassium homeostasis [12, 13] and in capillaries irrigating the pancreatic islets, regulating blood flow to the insulin-producing β-cells [10, 11].

Profound hypokalemia in SARS-CoV-2-infected patients has repeatedly been reported [14, 15], albeit at levels far from the current case, and a mechanistic model for this has been proposed [14]: ACE-2 disruption evoked by SARS-CoV-2 tilts the RAAS balance toward the angiotensin-II pathway, thereby enhancing aldosterone production. Excess aldosterone activates epithelial sodium channels, thus promoting urinary potassium excretion with attendant hypokalemia.

It is well known that severe hypokalemia may cause potentially lethal ventricular arrhythmias and respiratory muscular dysfunction, calling for increased vigilance of hypokalemia as a serious but easily diagnosed and treatable complication of SARS-CoV-2 infection, which has hitherto been somewhat neglected.

A vascular hallmark and common denominator of both SARS-CoV-2 infection [12] and unfolding of type 1 diabetes [16-18] is microangiopathy. The disruption of ACE-2 that occurs in host cells during SARS-CoV-2 infection results in greatly elevated levels of angiotensin-II [12], a potent vasoconstrictor, which in animal models has been shown to constrict blood flow to the pancreatic islets and thereby reduce insulin secretion [10, 11]. Recently it was also shown that the SARS-CoV-2 spike protein can induce pericyte and microvascular dysfunction in human pancreatic islets [16-18]. Moreover, SARS-CoV-2 can infect and replicate in human pancreatic islets ex vivo [19]. In autopsy material, SARS-CoV-2 nucleocapsid protein was detected in the pancreas, with findings collectively suggesting the endocrine pancreas as a diabetogenic target for human SARS-CoV-2 infection [19].

An international network and database for reporting new cases of diabetes in COVID-19-positive patients (the CoviDiab Global Live Registry) has been established (https://covidiab.e-dendrite.com) and will hopefully in the not too distant future be able to conclusively answer whether or not there is a causal relationship between SARS-CoV-2 infection and new-onset diabetes.

There are many known causes of hypokalemia; a common one is gastrointestinal loss of potassium. However, this patient reported no vomiting or diarrhea prior to or during the hospital stay. Notwithstanding that high doses of insulin may also cause hypokalemia, we deem that the doses this patient received (5 U/hour [58 mU/kg/hour]) are too small to have significantly contributed to his profound and sustained hypokalemia.

If the the angiotensin-II/aldosterone-dependent mechanism—causing massive renal potassium loss—is operative in human pathology, this implies that severe hypokalemia by SARS-CoV-2 infection may be amenable to treatment with potassium-sparing drugs antagonizing the aldosterone receptor, such as spironolactone or eplerenone, whereas potassium supplementation (whether oral or IV) even in very high doses may be futile.

## Learning Points

- This paper sheds light on a possible connection between SARS-CoV-2 infection on the one hand and new-onset type 1 diabetes and profound hypokalemia on the other.
- Severe, sustained, treatment-resilient, and potentially lethal hypokalemia may occur in patients with SARS-CoV-2 infection.
- Overactivation by SARS-CoV-2 of the angiotensin-II/aldosterone pathway of the RAAS may result in massive renal loss of potassium, thus producing hypokalemia.
- Treatment of this hypokalemia with high doses of potassium may be futile due to continuous urinary potassium excretion but may respond to potassium-sparing drugs antagonizing the aldosterone receptor.

## Contributors

Both Å.S. and A.B. provided care for the patient and made individual contributions to authorship and reviewed and approved the final draft.

## Data Availability

Restrictions apply to the availability of some or all data generated or analyzed during this study to preserve patient confidentiality or because they were used under license. The corresponding author will on request detail the restrictions and any conditions under which access to some data may be provided.
